# Agreement between nonculprit stenosis follow-up iFR and FFR after STEMI (iSTEMI substudy)

**DOI:** 10.1186/s13104-020-05252-6

**Published:** 2020-09-01

**Authors:** Troels Thim, Matthias Götberg, Ole Fröbert, Robin Nijveldt, Niels van Royen, Sergio Bravo Baptista, Sasha Koul, Thomas Kellerth, Hans Erik Bøtker, Christian Juhl Terkelsen, Evald Høj Christiansen, Lars Jakobsen, Steen Dalby Kristensen, Michael Maeng

**Affiliations:** 1 Department of Cardiology, Aarhus University Hospital, Palle Juul-Jensens Boulevard 99, 8200 Aarhus N, Denmark; 2 Department of Cardiology, Skåne University Hospital, Malmö, Sweden; 3 Department of Cardiology, Örebro University, Örebro, Sweden; 4 Department of Cardiology, Radboud University Medical Center, Nijmegen, The Netherlands; 5 Cardiology Department, Hospital Prof. Doutor Fernando Fonseca, Amadora, Portugal

**Keywords:** ST-segment elevation myocardial infarction, Nonculprit stenosis, Fractional flow reserve, FFR, Instantaneous wave-free ration, iFR

## Abstract

**Objective:**

To evaluate agreement between instantaneous wave free ratio (iFR) and fractional flow reserve (FFR) for the functional assessment of nonculprit coronary stenoses at staged follow-up after ST-segment elevation myocardial infarction (STEMI).

**Results:**

We measured iFR and FFR at staged follow-up in 112 STEMI patients with 146 nonculprit stenoses. Median interval between STEMI and follow-up was 16 (interquartile range 5–32) days. Agreement between iFR and FFR was 77% < 5 days after STEMI and 86% after ≥ 5 days (p = 0.19). Among cases with disagreement, the proportion of cases with hemodynamically significant iFR and non-significant FFR were different when assessed < 5 days (5 in 8, 63%) versus ≥ 5 days (3 in 15, 20%) after STEMI (p = 0.04). Overall classification agreement between iFR and FFR was comparable to that observed in stable patients. Time interval between STEMI and follow-up evaluation may impact agreement between iFR and FFR.

## Introduction

In patients with ST-segment elevation myocardial infarction (STEMI), nonculprit coronary stenoses may be identified [1]. Staged evaluation of these stenoses may rely on angiography alone, instantaneous wave free ratio (iFR), fractional flow reserve (FFR) or other invasive or noninvasive methods [2].

In stable patients, the agreement between iFR and FFR in determining hemodynamic significance is approximately 80% [3]. In patients with recent myocardial infarction, baseline coronary blood flow may be increased while maximal hyperaemic blood flow may be decreased in both culprit and nonculprit arteries [2, 4]. Increased baseline flow will tend to lower iFR values leading to overestimation of the hemodynamic significance of stenoses, while decreased maximal hyperaemic blood flow will tend to increase FFR values leading to underestimation of the hemodynamic significance of stenosis [2, 4]. Thereby, recent myocardial infarction may affect the agreement between iFR and FFR compared to stable conditions.

In this iSTEMI (iFR in STEMI [5, 6]) substudy, we evaluated the agreement between iFR and FFR in nonculprit stenoses at staged follow-up after STEMI.

## Main text

### Methods

The iSTEMI study has been described [5, 6]. Briefly, we measured nonculprit stenosis iFR in the acute STEMI setting and nonculprit stenosis iFR and FFR at staged follow-up. Nonculprit stenosis FFR was not measured in the acute STEMI setting.

In the current substudy, we examined agreement between nonculprit stenosis follow-up iFR and FFR < 5 days after STEMI (corresponding to follow-up during usual index admission duration) and after ≥ 5 days (corresponding to follow-up after usual index admission duration) after STEMI. In iSTEMI, time interval between STEMI and follow-up evaluation was at the discretion of the treating physicians.

Patients were recruited at Aarhus University Hospital (Denmark), Skåne University Hospital (Sweden), Örebro University Hospital (Sweden), VU University Medical Center (The Netherlands), and Hospital Prof. Doutor Fernando Fonseca (Portugal).

Intervals between STEMI and follow-up are presented as median (interquartile range). We considered iFR < 0.90 and FFR ≤ 0.80 hemodynamically significant. Proportions were compared using a proportion calculator (Stata/IC 13.1).

## Results

The presented data are from 112 STEMI patients with completed follow-up iFR and FFR of 146 nonculprit stenoses [5]. We refer to the original publication for baseline patient data [5]. The median interval between STEMI and follow-up was 16 (5–32) days (Tables 1 and 2).

**Table 1:** Agreement between follow-up iFR and FFR of nonculprit lesions < 5 days after STEMI

	iFR ≥ 0.90	iFR < 0.90	Total
FFR > 0.80	11	5	16
FFR ≤ 0.80	3	16	19
Total	14	21	35

iFR, instantaneous wave free ratio. FFR, fractional flow reserve. STEMI, ST-segment elevation myocardial infarction. Follow-up interval: 2 (1–3) days

**Table 2:** Agreement between follow-up iFR and FFR of nonculprit lesions ≥ 5 days after STEMI

	iFR ≥ 0.90	iFR < 0.90	Total
FFR > 0.80	60	3	63
FFR ≤ 0.80	12	36	48
Total	72	39	111

iFR, instantaneous wave free ratio. FFR, fractional flow reserve. STEMI, ST-segment elevation myocardial infarction. Follow-up interval: 28 (12–34) days

In patients re-evaluated < 5 days after STEMI, there was classification agreement between iFR and FFR in 77% of nonculprit stenoses. When staged evaluation was performed ≥ 5 days after STEMI, classification agreement was 86% (p = 0.19). Among cases with classification disagreement, the proportions of cases with hemodynamically significant iFR and non-significant FFR were different when assessed < 5 days (5 in 8, 63%) versus ≥ 5 days (3 in 15, 20%) after STEMI (p = 0.04). Individual values of follow-up iFR and FFR in patients with disagreement on nonculprit stenosis significance between iFR and FFR are presented in the Additional file 1.

## Discussion

The time interval between myocardial infarction and normalisation of baseline coronary blood flow and maximal hyperaemic blood flow remains uncertain and may differ between patients. The impact of these temporary blood flow changes on iFR may resolve before the impact on FFR and the impact on iFR may need more than 2 weeks to resolve [4–8].

In the current study, the time interval between STEMI and nonculprit stenosis evaluation did not impact the overall agreement between iFR and FFR. However, among cases with disagreement between follow-up iFR and FFR, iFR was more likely than FFR to indicate hemodynamic significance < 5 days after STEMI whereas FFR was more likely than iFR to indicate hemodynamic significance after ≥ 5 days. The observations after ≥ 5 days probably resemble observations in stable conditions more closely, i.e., FFR is more often significant than iFR with similar outcomes of revascularisation guided by iFR and FFR [9, 10]. Within < 5 days after STEMI, both iFR and FFR may be affected, but in opposite directions, and the optimal method for nonculprit stenosis evaluation in this setting remains undetermined [4–8]. Also, the optimal time point for making this assessment remains to be established [2]. Different methods can be applied taking timing and potential bias of the used method in relation to timing into consideration [2]. In the acute or subacute setting, baseline flow may be increased and hyperemic flow may be decreased which may yield decreased iFR (overestimation of stenosis significance) and increased FFR (underestimation of stenosis significance) and these changes are expected to normalize over time although the time frame for this normalization is undetermined and may vary between patients [4–6].

In conclusion, in staged nonculprit stenosis evaluation after STEMI, iFR and FFR has an overall agreement that is comparable to that observed in stable patients. However, the time interval between STEMI and follow-up evaluation may impact agreement between iFR and FFR.

## Limitations

The used cutoffs for iFR and FFR are based on previous studies and current clinical practice and derived from patients in stable condition. There was no clearly defined gold standard for ischemia detection. The cohort should ideally be a consecutive cohort, however, not all patients eligible patients were included during the study period. Without FFR in the acute setting, there is no information on the change in FFR.

The observed iFR values were clustered around the cut-off for iFR. The distribution of iFR and FFR values may affect the agreement between the two methods. There was no data on microvascular obstruction or infarct size in these patients.

## Supplementary information

**Additional file 1.** Individual values of follow-up iFR and FFR in patients with disagreement on nonculprit stenosis significance between iFR and FFR.

## Data Availability

The datasets used and/or analysed during the current study are available from the corresponding author on reasonable request.

## Abbreviations

FFR: Fractional flow reserve
iFR: Instantaneous wave-free ratio
STEMI: ST-segment elevation myocardial infarction
