# Supplementary material for: Agreement between nonculprit stenosis follow-up iFR and FFR after STEMI (iSTEMI substudy)
Source: BMC Res Notes. 2020 Sep 1;13:410. doi: 10.1186/s13104-020-05252-6 (PMC7466494; doi:10.1186/s13104-020-05252-6)
Supplement: Supplementary file 1 — Additional file 1. Individual values of follow-up iFR and FFR in patients with disagreement on nonculprit stenosis significance between iFR and FFR. [file 13104_2020_5252_MOESM1_ESM.docx]

Supplement to

**Agreement between nonculprit stenosis follow-up iFR and FFR after STEMI (iSTEMI substudy)**

Troels Thim^1^, MD, PhD; Matthias Götberg^2^, MD, PhD; Ole Fröbert^3^, MD, PhD; Robin Nijveldt^4^, MD, PhD; Niels van Royen^4^, MD, PhD; Sergio Bravo Baptista^5^, MD, PhD; Sasha Koul^2^, MD, PhD; Thomas Kellerth^3^, MD; Hans Erik Bøtker^1^, MD, DMSc; Christian Juhl Terkelsen^1^, MD, PhD, DMSc; Evald Høj Christiansen^1^, MD, PhD; Lars Jakobsen^1^, MD, PhD; Steen Dalby Kristensen^1^, MD, DMSc; Michael Maeng^1^, MD, PhD

Individual values of follow-up iFR and FFR in patients with disagreement on nonculprit stenosis significance between iFR and FFR.

**Follow-up iFR and FFR of nonculprit lesions <5 days after STEMI.**

Significant stenosis by iFR and not by FFR (n=5).

| iFR | FFR |
| --- | --- |
| 0.89 | 0.85 |
| 0.80 | 0.91 |
| 0.89 | 0.82 |
| 0.84 | 0.81 |
| 0.88 | 0.84 |

Significant stenosis by FFR and not by iFR (n=3).

| iFR | FFR |
| --- | --- |
| 0.91 | 0.80 |
| 0.90 | 0.79 |
| 0.96 | 0.75 |

**Follow-up iFR and FFR of nonculprit lesions ≥5 days after STEMI.**

Significant stenosis by iFR and not by FFR (n=3).

| iFR | FFR |
| --- | --- |
| 0.89 | 0.83 |
| 0.83 | 0.83 |
| 0.89 | 0.87 |

Significant stenosis by FFR and not by iFR (n=12).

| iFR | FFR |
| --- | --- |
| 0.94 | 0.75 |
| 0.91 | 0.78 |
| 0.91 | 0.76 |
| 1.00 | 0.75 |
| 0.96 | 0.76 |
| 0.93 | 0.75 |
| 0.94 | 0.78 |
| 0.94 | 0.77 |
| 0.92 | 0.60 |
| 0.91 | 0.75 |
| 0.95 | 0.76 |
| 0.90 | 0.75 |
